# snRNA-seq reveals subcutaneous white adipose tissue remodeling upon return to thermoneutrality after cold stimulation

**DOI:** 10.3389/fcell.2025.1578180

**Published:** 2025-05-22

**Authors:** Yusha Yang, Guanyu Zhang, Ting Yi, Shuran Yang, Shuai Wu, Yongqiang Zhang, Li Zhang, Xi Li, Xiuxuan Wu, Jun Li, Danfeng Yang

**Affiliations:** ^1^ School of Public Health, The Key Laboratory of Environmental Pollution Monitoring and Disease Control, Ministry of Education, Guizhou Medical University, Guiyang, China; ^2^ Academy of Military Medical Sciences, Academy of Military Sciences, Tianjin, China; ^3^ School of Chinese Materia Medica, Tianjin University of Traditional Chinese Medicine, Tianjin, China

**Keywords:** single-nucleus RNA sequencing, thermoneutrality, subcutaneous white adipose tissue, plasticity, cold stimulation

## Abstract

**Introduction:**

Cold stimulation induces browning of subcutaneous white adipose tissue (sWAT), making it a prime target for treating obesity and metabolic disorders. However, this remodeling is reversible: upon return to thermoneutrality (rewarming), sWAT whitens and loses its enhanced metabolic functions. Given the limited understanding of the microscopic dynamic changes and underlying mechanisms during this process, we established a temporally dynamic mouse model spanning the entire period from cold stimulation to the return to thermoneutrality, with inguinal sWAT (iWAT) selected as the study subject.

**Methods:**

Based on preliminary data demonstrating stabilization in iWAT histology, expression levels of key thermogenic proteins, and the bulk transcriptome, we selected the two-week time point after the return to thermoneutrality for detailed analysis. Subsequently, we employed single-nucleus RNA sequencing (snRNA-seq) to comprehensively characterize iWAT cellular dynamics during cold stimulation and the subsequent two-week period after the return to thermoneutrality.

**Results:**

Our findings revealed that while iWAT phenotypically reverts to a white state after 2 weeks of rewarming, as evidenced by structural, functional, and bulk transcriptomic characteristics, significant cold-induced molecular and cellular signatures persist. Specifically, we observed altered differentiation trajectories in both adipose stem and progenitor cells (ASPCs) and adipocytes, suggesting dedifferentiation and reprogramming tendencies. Furthermore, the ANGPTL signaling pathway, activated in thermogenic adipocyte subpopulation A3 during cold stimulation, remained active and influenced cell-cell communication even after the loss of thermogenic capacity.

**Discussion:**

hese findings provide novel insights into elucidating the complex cellular and molecular mechanisms underlying the temperature-dependent plasticity of iWAT, and suggest that the ANGPTL signaling pathway may play a potential role in maintaining the white phenotype of iWAT after withdrawal from cold stimulation.

## 1 Introduction

White adipose tissue (WAT), which is widely distributed throughout the human body, serves as the primary energy storage organ. It can be broadly categorized into sWAT and visceral WAT (vWAT) based on anatomical location. sWAT, constituting approximately 80% of total body fat, exhibits a high degree of plasticity (Wajchenberg, 2000). Recent research has demonstrated that sWAT dynamically regulates physiological processes such as thermogenesis and energy metabolism in response to changes in nutritional demands and environmental factors, thus holding immense potential for the prevention and treatment of metabolic diseases (Bartelt and Heeren, 2014; Hanssen et al., 2015; Cohen and Kajimura, 2021). As a key player in maintaining systemic homeostasis, sWAT is typically distinguished from brown adipose tissue (BAT) by the structure of its adipocytes. Unlike brown adipocytes, which contain multilocular lipid droplets and abundant mitochondria, white adipocytes within sWAT possess a single large unilocular lipid droplet and a limited number of mitochondria. This characteristic facilitates efficient storage of excess energy in the form of triglycerides (Hwang and Kim, 2019). However, this state undergoes a profound phenotypic shift upon cold stimulation. sWAT is stimulated to generate beige adipocytes, exhibiting remarkable structural and functional convergence with brown adipocytes. These beige adipocytes possess the capacity to dissipate surplus energy as heat, through mechanisms both dependent on and independent of uncoupling protein 1 (UCP1), thereby augmenting cold tolerance and mitigating metabolic derangements (Cohen and Kajimura, 2021). Notably, upon return to thermoneutral conditions, beige adipocytes undergo a phenotypic reversion, adopting a white adipocyte-like morphology and function. Intriguingly, recent investigations have revealed that these “whitened” beige adipocytes retain a cellular memory of prior cold stimulation, facilitating an accelerated response to subsequent cold challenges and a more rapid reinstatement of the thermogenic state (Roh et al., 2018). Therefore, understanding how to maintain and consolidate the thermogenic capacity of beige adipocytes within sWAT, or to enhance their responsiveness to environmental stimuli, may hold significant implications for improving metabolic health in individuals living in thermoneutral environments where WAT may not receive sufficient stimulation for sustained thermogenesis. The remarkable plasticity of sWAT in response to temperature fluctuations is attributed to its heterogeneous cellular composition, including mature adipocytes and a stromal vascular fraction (SVF) comprising mesenchymal stem cells, immune cells, and endothelial cells (Wang et al., 2022). While extensive cellular and structural remodeling drives sWAT adaptation during cold stimulation and subsequent return to thermoneutrality, the underlying mechanisms, particularly those governing the post-cold stimulation phase, remain poorly understood. This knowledge gap underscores the need for comprehensive investigations leveraging advanced technologies such as single-cell transcriptomics to dissect the complex cellular and molecular dynamics of sWAT remodeling.

To elucidate the dynamic remodeling of sWAT following cold stimulation and subsequent return to thermoneutrality, a temporally dynamic mouse model was established under alternating 4°C (cold stimulation) and 30°C (thermoneutrality) conditions. snRNA-seq was performed on iWAT from mice at four key time points spanning cold stimulation through return to thermoneutrality, based on tissue morphology, expression levels of the key thermogenic protein, and bulk-transcriptomic profiles. Our comprehensive analysis provides an in-depth characterization of the diverse categories of iWAT alterations induced by recovery from thermal challenge after cold stimulation, encompassing reversible, irreversible, transient, and persistent changes across diverse cellular populations. These changes were observed in cell type composition, gene expression programs, and intercellular communication networks. Of particular interest, we observed that ASPCs and adipocyte subpopulations exhibited altered differentiation and developmental trajectories upon return to thermoneutrality, diverging from those initially shaped by cold stimulation. Furthermore, intercellular communication analysis underscored the central role of adipocytes in orchestrating iWAT responses throughout the entire process, highlighting their extensive interactions with other resident cell types. Finally, we identified angiopoietin-like 4 (*Angptl4*) as a putative key factor in mediating the reversion of beige adipocytes to a white adipocyte-like phenotype, contributing to the maintenance of this “whitened” state and potentially playing a role in the preservation of inter-organ metabolic homeostasis.

## 2 Materials and methods

### 2.1 Key resources and table

**Table:** 

Reagent or resource	Category	Source	Identifier or version
Rabbit anti-UCP1 antibody	Antibodies	Servicebio	Cat# GB112174
Rabbit anti-PLIN1 antibody	Antibodies	CST	Cat# 9349T
BSA	Chemicals, peptides	Servicebio	Cat# GC305010
4% PFA Fix Solution	Chemicals, peptides	Biosharp	Cat# 23199846
RNA Later™	Chemicals, peptides	Beyotime	Cat# R0118-100 mL
Rabbit anti-UCP1 antibody	Antibodies	Servicebio	Cat# GB112174
NovaSeq™ 6000 v1.5 reagent kits	Critical commercial assays	Illumina	Cat# 20028312
Shbio Nuclei Isolation Kit	Critical commercial assays	SHBIO	Cat# 52009–10
Mouse: male C57BL/6J	Other	Vital River Laboratory	SCXK(Beijing) 2021–0006

### 2.2 Animals

Eight-week-old male C57BL/6J mice (Vital River Laboratory Animal Technology Company, Beijing, China) were acclimated at 30°C for 10 days, cold-exposed at 4°C for 2 weeks, and then returned to 30°C for 3 days to 6 weeks. Mice were housed under standard conditions with a 12 h/12 h light/dark cycle and free access to food and water. Following anesthesia, iWAT was collected for bulk RNA sequencing (n = 21), histological analysis (n = 21), and SnRNA sequencing (n = 24). All animal experimental procedures were approved by the Committee on the Ethics of Animal Experiments of the Academy of Military Medical Science (Approval Code: AMMS-04-2022-027. Date of approval: 2022.07.04).

### 2.3 Histological and immunofluorescent staining

Following overnight fixation in 4% paraformaldehyde, iWAT samples are embedded in paraffin and sectioned. Sections are stained with hematoxylin and eosin (HE) and imaged using an inverted microscope (Laica, Germany) to assess adipocyte morphology. For immunofluorescent staining (IF), iWAT sections undergo deparaffinization, rehydration, and antigen retrieval. Endogenous peroxidase activity is blocked, followed by incubation with primary antibodies against UCP1 (1:2000, GB112174, Servicebio) and PLIN1 (1:200, 9349T, CST). Sections are then incubated with secondary antibodies conjugated to HRP (1:500, GB23204, Servicebio) and Alexa Fluor 488 (1:400, GB25303, Servicebio). Images are acquired using an inverted microscope (Laica, Germany).

### 2.4 Single-nucleus RNA sequencing

Frozen iWAT samples are processed for nuclear isolation using the Shbio Nuclei Isolation Kit (SHBIO, Shanghai, China) according to the manufacturer’s instructions. All procedures are performed on ice. Single-nucleus suspensions are loaded onto the Chromium Single Cell 3′Reagent Kit v3 platform (10x Genomics, United States) and libraries are prepared according to the manufacturer’s protocol. Sequencing is performed on an Illumina NovaSeq 6000 sequencing system (paired-end multiplexing run, 150 bp). The workflow includes assessing nuclei concentration and quality, encapsulating individual nuclei in gel beads-in-emulsion (GEMs) with unique barcodes, reverse transcribing nuclear RNA into cDNA within each GEM followed by cleanup, amplifying cDNA, and constructing libraries for sequencing on the Illumina NovaSeq platform.

### 2.5 Bulk RNA sequencing and analysis

Sequencing libraries were generated using the NovaSeq 6000 S4 Reagent Kit v1.5 for Illumina. RNA and library quality were assessed using an Agilent Bioanalyzer 2100 system and Qubit 3. The sequencing was performed on an Illumina platform (150 bp paired-end reads). Reads were mapped to the mouse reference transcriptome (Ensembl; Mus_musculus. GRCm38.91) using HISAT (2.0.5). Differential gene expression (DEG) analysis was performed in R (4.3.1) using DESeq2 (1.40.2). Genes with an adjusted p-value <0.05 and |log2 fold change| > 1 were considered differentially expressed.

### 2.6 snRNA-seq data Preprocessing

Raw sequencing data were processed using Cell Ranger (6.01) with the mouse reference genome (Ensembl; Mus_musculus.GRCm38.91) to generate feature-barcode matrices. These matrices were loaded into Seurat (5.0.3) in R (4.3.2) and analyzed following the standard Seurat workflow (Hao et al., 2023). Low-quality cells (mitochondrial DNA >20%, ribosomal DNA >15%, unique UMI count <200 or >6000) were removed, and potential doublets were identified and excluded using DoubletFinder (McGinnis et al., 2019). The remaining data were normalized, scaled, and integrated using Harmony (Korsunsky et al., 2019). Clustering was performed using the Louvain algorithm, and clusters were visualized using UMAP. Cell types were annotated based on marker genes identified with the FindAllMarkers function (Wilcoxon rank sum test, min.pct = 0.1, logfc.threshold = 0.25).

### 2.7 Gene enrichment analysis

Kyoto Encyclopedia of Genes and Genomes (KEGG) and Gene Ontology (GO) enrichment analyses were performed using the ClusterProfiler package in R (4.3.2) (Yu et al., 2012).

### 2.8 Trajectory inference and RNA velocity

Trajectory inference was performed using Monocle (2.14.0) in R (4.3.2). Briefly, a CellDataSet object was created, and highly variable genes associated with pseudotime were identified using the dispersionTable function (Qiu et al., 2017). Cell trajectories were visualized using plot_cell_trajectory. RNA velocity analysis was performed using scVelo (0.3.2) in Python (3.8) with default parameters (Bergen et al., 2020). Briefly, count matrices were normalized to the median total molecules across cells. The top 2000 highly variable genes were selected for spliced and unspliced mRNA analysis. First- and second-order moments were calculated for each cell using its 30 nearest neighbors for velocity estimation.

### 2.9 Cell-cell communication inference

Ligand-receptor interactions were analyzed using CellChat (1.6.1) in R (4.3.2) (Jin et al., 2021). Cells from four time points (T0, T1, T2, T4) were aggregated by cell type (11 total) for CellChat analysis using the mouse ligand-receptor database and default parameters (with min cells set to 10). Communication networks were visualized using the netVisual_circle function, with each communication defined as a unique combination of sender cell type, ligand, receptor, and receiver cell type. The contribution of signaling pathways and ligand-receptor pairs to outgoing and incoming signals for each cell group was visualized using the netAnalysis_signalingRole_heatmap function. Significant interactions between selected cell groups across different conditions were visualized using the netVisual_bubble function.

## 3 Results

### 3.1 iWAT achieves stable remodeling after dynamic changes upon return to thermoneutrality following cold stimulation

**FIGURE 1:**
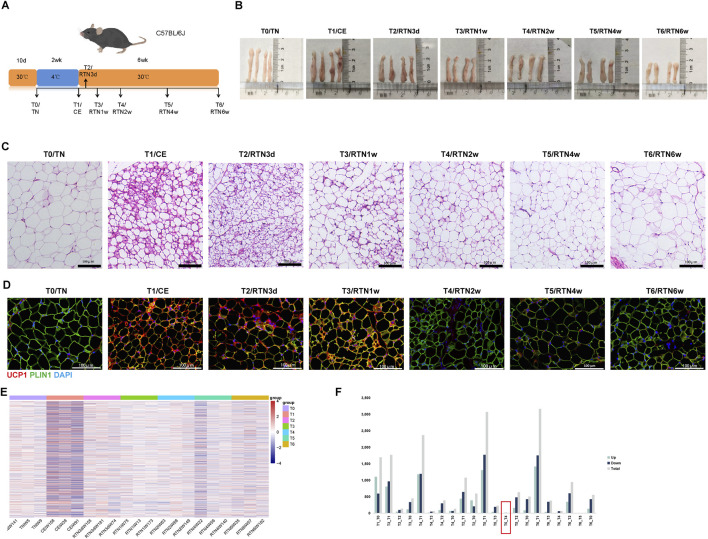
iWAT Achieves Stable Remodeling After Dynamic Changes Upon Return to Thermoneutrality Following Cold Stimulation **(A)** A mouse model of cold stimulation followed by a return to thermoneutrality was established. Eight-week-old male C57BL/6J mice were initially housed at 30°C for 10 days (T0/TN) followed by cold stimulation at 4°C for 2 weeks (T1/CE), and subsequently returned to 30°C for 3 days (T2/RTN3d), 1 week (T3/RTN1w), 2 weeks (T4/RTN2w), 4 weeks (T5/RTN4w), or 6 weeks (T6/RTN6w). iWAT was collected at each time point (T0-T6) for analysis. **(B)** Gross morphology of iWAT from mice at T0-T6. **(C)** HE stains of iWAT sections from mice at T0-T6 (n = 21). Scale bar, 100 μm. **(D)** IF staining of iWAT sections for perilipin 1 (PLIN1) and uncoupling protein 1 (UCP1) in mice at T0-T6. Scale bar, 100 μm. (n = 3 per group). **(E)** Summary of differentially expressed genes (DEGs) identified by bulk RNA sequencing analysis of iWAT (n = 21). **(F)** Heatmap showing the expression of all DEGs in iWAT at T0-T6.

iWAT exhibits remarkable plasticity, undergoing browning in response to cold stimulation. This process involves structural and functional remodeling, leading to the emergence of beige adipocytes with thermogenic and metabolic regulatory roles. However, upon return to thermoneutrality, this balance is disrupted, and beige adipocytes revert to a white phenotype. Interestingly, subsequent cold stimulation will trigger a more rapid activation of thermogenic programs, suggesting a memory effect (Roh et al., 2018; Lundgren et al., 2023). To investigate the dynamic changes in iWAT cellular heterogeneity during the transition from cold stimulation back to thermoneutrality, and to explore the mechanisms underlying this memory effect, we established a mouse model involving an initial thermoneutral acclimation (30°C, 10 days), followed by cold stimulation (4°C, 2 weeks), and finally a return to thermoneutrality for varying durations (30°C, 3 days to 6 weeks) (Figure 1A). Consistent with previous reports, 2 weeks of continuous low-temperature stimulation caused iWAT to acquire a reddish-brown appearance. Upon return to thermoneutrality, the tissue gradually regained its white appearance, and its volume changed dynamically (Figure 1B). Meanwhile, this trend of change was also observed in tissue morphology and the expression of thermogenic proteins. HE stains and IF analysis of iWAT from each group revealed that sustained cold stimulation induced a shift in lipid droplet morphology from large, unilocular droplets to smaller, multilocular droplets. Concurrently, the expression of the thermogenic protein UCP1 had significantly increased. However, upon return to a thermoneutral environment, these changes gradually reversed, trending towards the initial state observed prior to cold stimulation (T0/TN). Notably, after 2 weeks in the thermoneutral environment (T4/RTN2w), the histologically visible small multilocular lipid droplets had disappeared, while UCP1 expression had almost completely returned to pre-cold stimulation baseline levels (Figures 1C,D). Furthermore, we performed bulk RNA sequencing on the iWAT collected and conducted DEG analysis using DESeq2 to compare all groups. This analysis revealed that the overall differential gene expression profile in iWAT, which was significantly altered by cold stimulation, tended to revert to its pre-cold stimulation state upon return to thermoneutrality for 2 weeks (T4) (Figure 1E). Notably, the fewest differentially expressed genes were observed between T4 and T5 (return to thermoneutrality for 4 weeks), suggesting that the remodeling within iWAT may have stabilized by 2 weeks after returning to thermoneutral conditions (Figure 1F). Therefore, based on these macro-level findings, we selected the two-week post-return to thermoneutrality time point for detailed analysis. This critical juncture in the reversal of cold-induced tissue remodeling allowed us to comprehensively investigate the dynamic microenvironmental changes and molecular regulatory mechanisms underlying both the remodeling process during sustained cold stimulation and the gradual ‘recovery’ phase upon reacclimation to thermoneutral conditions in iWAT.

### 3.2 SnRNA-seq reveals dynamic remodeling of cell composition and gene expression profiles in iWAT upon return to thermoneutrality after cold stimulation

**FIGURE 2:**
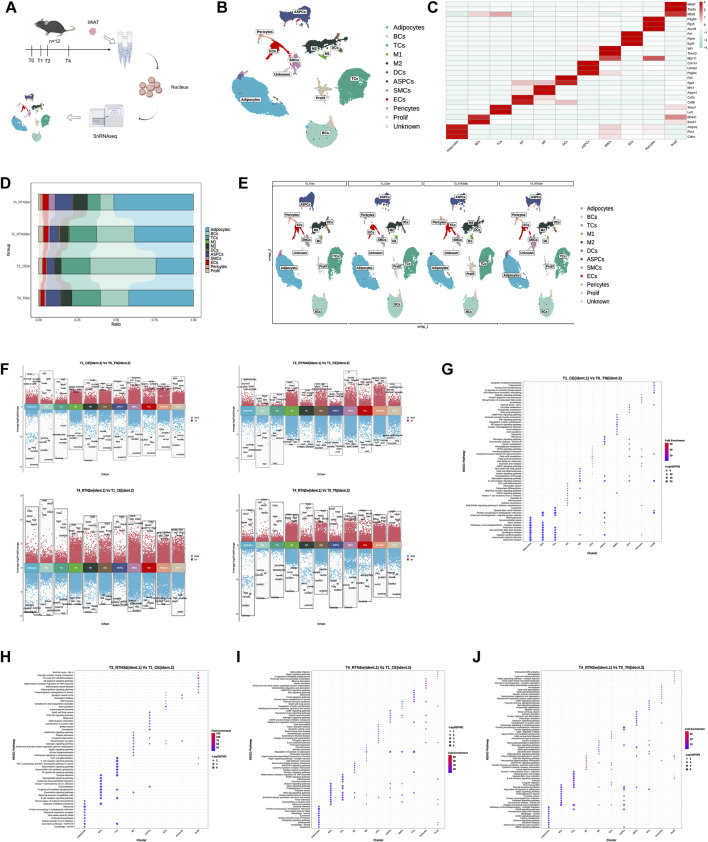
SnRNA-seq Reveals Dynamic Remodeling of Cell Composition and Gene Expression Profiles in iWAT Upon Return to Thermoneutrality After Cold Stimulation **(A)** Schematic overview of the snRNA-seq experimental workflow. Nuclei are isolated from the iWAT of mice at T0, T1, T2, and T4. Sequencing is performed using the 10x Genomics platform, followed by bioinformatic analysis. **(B)** UMAP of cells (n = 97,666) identified in iWAT across the four time points. Cell clusters include adipocytes, B lymphocytes (BCs), T lymphocytes (TCs), M1 macrophages (M1), M2 macrophages (M2), dendritic cells (DCs), adipose stem and progenitor cells (ASPCs), pericytes, endothelial cells (ECs), smooth muscle cells (SMCs), proliferation-related immune cells (Prolif), and an unknown cell type. **(C)** Heatmap showing the scaled average expression of highly variable genes and canonical markers across iWAT cell clusters. **(D,E)** Percent bar graphs and UMAPs showing the changes in the composition of the 11 cell types in iWAT under T0, T1, T2, and T4. **(F)** Volcano plots showing upregulated and downregulated genes in 11 clusters in the comparison group (ident.1) relative to the control group (ident.2). Upregulated genes (average logFC >0 and adjusted p-value <0.05) are shown in red, while downregulated genes (average logFC <0 and adjusted p-value <0.05) are shown in blue. **(G–J)** KEGG pathway enrichment analysis of upregulated genes in each cluster in the comparison group (ident.1) relative to the control group (ident.2). The top 10 pathways are shown for comparison.

To investigate the dynamic cellular and molecular changes in iWAT during the transition from cold stimulation back to thermoneutrality, we collected from mice at four time points: T0 (thermoneutrality, TN), T1 (cold stimulation, CE), T2 (3 days after return to thermoneutrality, RTN3d), and T4 (2 weeks after return to thermoneutrality, RTN2w) (Figure 1A), and single-nucleus suspensions were prepared according to the 10X Genomics protocol to perform snRNA-seq (Figure 2A). Sequenced data were processed with Cell Ranger and analyzed in R using the Seurat package. Following quality control and data integration (see Methods), 97,666 high-quality cells were retained for downstream analysis. By applying an unsupervised clustering method within Seurat, 12 cell clusters were identified in the integrated dataset and visualized through the application of Uniform Manifold Approximation and Projection (UMAP) (Figure 2B). Based on marker gene expression, these clusters were identified as adipocytes (*Plin1* ^*+*^), B lymphocytes (*Ms4a1* ^*+*^, BCs), T lymphocytes *(Lef1* ^*+*^, TCs), M1 macrophages (*Cd86* ^*+*^, M1), M2 macrophages (*Mrc1* ^*+*^, M2), dendritic cells (*Flt3* ^*+*^, DCs), ASPCs (*Pdgfrα* ^*+*^), pericytes (*Pdgfrβ* ^*+*^), endothelial cells (*Ptprb* ^*+*^, ECs), smooth muscle cells (*Mhy11* ^*+*^, SMCs), proliferation-related immune cells (*Top2a* ^*+*^, Prolif), and an undefined cluster designated as “unknown” following differential gene expression and pathway analyses (Figure 2C).

Comparison of cell type proportions across the four time points revealed that cold stimulation induced alterations in the cellular composition of iWAT. However, upon return to thermoneutrality, most cell type proportions gradually reverted to near-initial (T0) levels (Figures 2D,E). For instance, the proportions of adipocytes, macrophages (M1 and M2), and ASPCs decreased during cold stimulation but gradually increased upon rewarming. In contrast, TCs and BCs showed an initial increase followed by a gradual decline. Interestingly, although these cellular proportion trends aligned with phenotypic changes observed in iWAT (Figures 1C,D) — showing a distinct “recovery” pattern during thermoneutral readaptation, the cellular composition at 2 weeks post-recovery (T4) still differed discernibly from the pre-cold-stimulation baseline (Figures 2D,E). This indicates that cold stimulation induces lasting changes in the iWAT microenvironment, even after the mice have re-acclimated to a thermoneutral environment. These changes potentially prime the tissue for an accelerated response to subsequent cold challenges.

Finally, DEG analysis revealed extensive transcriptomic remodeling across cell clusters during the transition from cold stimulation back to thermoneutrality (Figure 2F). Further KEGG pathway analysis of differentially expressed genes during cold stimulation (T1 vs. T0) and rewarming (T2/T4 vs. T1) highlighted distinct functional responses. In adipocytes, genes upregulated during cold stimulation were enriched in thermogenic pathways. However, this enrichment was lost upon return to thermoneutrality. Instead, upregulated genes at 3 days of rewarming were enriched in pathways related to carbohydrate metabolism and genetic information processing, while those at 2 weeks were enriched in cellular processes like mitophagy, autophagy, and apoptosis. Immune cells, such as B cells and macrophages, displayed enrichment for both immune response and energy metabolism pathways during cold stimulation, including fatty acid metabolism, PPAR signaling, and AMPK signaling. Upon rewarming, the enrichment shifted towards immune response and signal transduction, with a reduction in the number of enriched pathways. ASPCs, ECs, pericytes, and SMCs, which are involved in adipogenesis and angiogenesis, exhibited enrichment for various metabolic pathways, cell growth and differentiation, and thermogenesis during cold stimulation. Upon rewarming, the number and diversity of enriched pathways decreased, with a shift away from metabolism (Figures 2G–J). Furthermore, a comparison of gene expressions at 2 weeks after rewarming (T4) with the initial thermoneutral state (T0) identified 109952 differentially expressed genes across 11 clusters, including 61059 upregulated genes. Enrichment analysis of these upregulated genes revealed a significant enrichment of metabolic pathways in most cell types. Importantly, these results contrasted with our aforementioned observations regarding iWAT appearance, histology, and bulk RNA-seq (where returning to thermoneutrality for 2 weeks largely restored cold stimulation-induced remodeling in iWAT to pre-cold stimulation levels). This suggests that the macroscopic phenotypic recovery in iWAT might be “deceptive,” and that the dynamic remodeling induced by cold stimulation and subsequent return to thermoneutrality leads iWAT into a new state, distinct from its initial condition. Critically, the divergence between these two states may hold the key to developing strategies to consolidate high-level thermogenic and metabolic functions in iWAT.

### 3.3 Shifting subpopulation differentiation trajectories of ASPCs in iWAT upon return to thermoneutrality after cold stimulation

**FIGURE 3:**
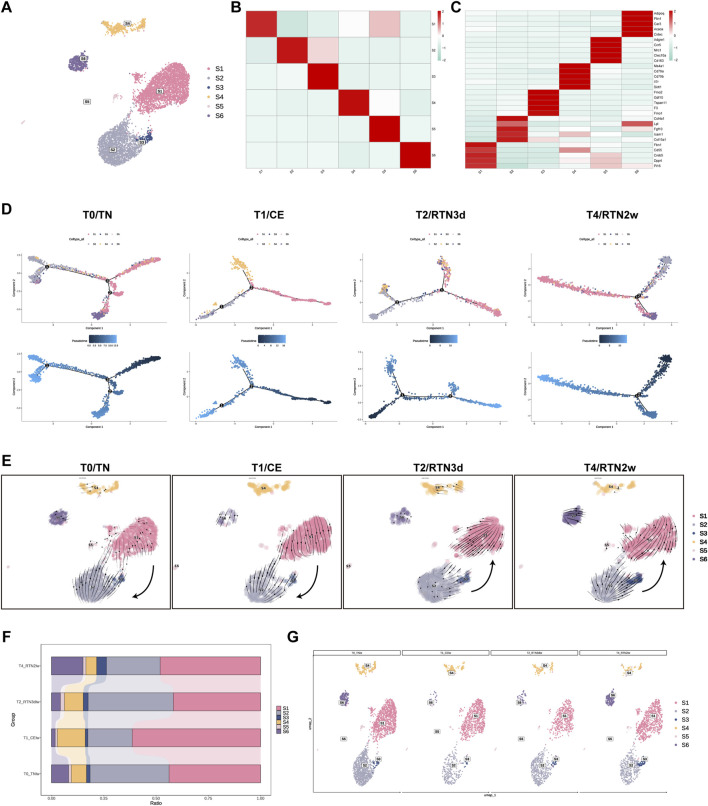
Shifting Subpopulation Differentiation Trajectories of ASPCs in iWAT Upon Return to Thermoneutrality After Cold Stimulation. **(A)** UMAP of subpopulations (S1-S6) identified within the ASPCs population following re-clustering. **(B)** Heatmap showing the average scaled gene module scores for the top 50 most significant genes within each ASPCs subcluster (S1-S6). **(C)** Heatmap showing the scaled average expression of highly variable genes and canonical markers across ASPC subclusters (S1-S6). **(D)** The trajectory inference of ASPCs subpopulations (S1-S6) across the T0, T1, T2, and T4. **(E)** The RNA velocity of ASPCs subpopulations (S1-S6) across the T0, T1, T2, and T4. **(F,G)** Percent bar graphs and UMAPs showing the changes in the composition of ASPC subpopulations (S1-S6) under T0, T1, T2, and T4.

ASPCs constitute a crucial component of the SVF within adipose tissue (AT), where they exert a pivotal influence on AT function and plasticity. ASPCs not only govern the composition of mature adipocytes but also exhibit a remarkable degree of heterogeneity. Recent investigations have revealed the existence of at least three distinct ASPC subpopulations, each characterized by unique properties such as proliferative capacity and adipogenic potential (Schwalie et al., 2018; Merrick et al., 2019; Sarvari et al., 2021; Shao et al., 2021; Liu et al., 2023). To elucidate the degree of cellular heterogeneity within ASPCs during the dynamic remodeling of iWAT elicited by cold stimulation and subsequent return to thermoneutrality, and to further delineate the contributions of distinct ASPCs subtypes to the maintenance of organismal homeostasis, we conducted a subclustering analysis. This analysis identified 6 distinct subpopulations (S1-S6), each characterized by unique gene expression profiles (Figures 3A,B). Annotation of these subclusters was performed based on the expression of highly variable genes, drawing upon previous studies for comparative analysis (Figure 3C). S1, S2, and S3 exhibited transcriptional profiles analogous to the DPP4+/adipocyte stem cell (ASC), ICAM1+/preadipocyte (preA), and CD142+/amphiregulin cell (Areg), respectively—as delineated by Merrick’ team (Merrick et al., 2019) and Liu’ team (Liu et al., 2023). Specifically, S1 highly expressed genes such as *Dpp4*, *Cd55*, and *Pi*, characteristic of ASC. S2 was characterized by the expression of *Icam1*, collagen, extracellular matrix (ECM) remodeling factors like *Col4a1*, *Col4a2*, and *Fgf10*, and the adipogenic marker *Lpl*, representing preA. S3 expressed *F3/Cd142*, *Gdf10*, and the anti-adipogenic genes *Fmo2* and *Tgfbi*, consistent with Areg. Among the newly identified clusters (S4, S5, and S6), S4 and S5 were distinguished by the expression of lymphoid and myeloid immune cell markers, respectively, including *Il7r*, *Ms4a1*, and *Mrc1*. This observation suggests a potential involvement of these subpopulations in immune modulation within the ASPCs compartment, aligning with the findings of Xie et al. (2023). Notably, S6 exhibited elevated expression of adipocyte markers such as *Adipoq*, *Plin1*, and *Cidec*, indicating a transitional state in adipocyte differentiation, characterized by ongoing lipid accumulation and intermediate phenotypic features between preadipocytes and mature adipocytes.

To determine the lineage relationships among ASPC subpopulations, we employed Monocle 2 to perform trajectory inference analysis across the four experimental time points. Consistent with previous studies (Burl et al., 2018; Liu et al., 2023) using cold stimulation models, our analysis revealed that S1 possessed the capacity to differentiate into both S2 and S3, exhibiting distinct directional biases before and after cold stimulation. Furthermore, S6 occupied the terminal position within the inferred differentiation trajectory, while the immune-associated subclusters, S4 and S5, were positioned along the entire trajectory, suggesting their integral involvement in the differentiation process (Figure 3D; Supplementary Figure S1A). Intriguingly, our analysis unveiled a dynamic shift in the differentiation trajectory between S1 and S2/S3 during the rewarming phase, characterized by a reversion of S2/S3 towards S1, which implies that two-week reacclimation to thermoneutrality drives the dedifferentiation of S2/S3 (primed for adipocyte commitment) back to multipotent S1. This observation was further supported by RNA velocity analysis conducted using Scvelo, which independently confirmed this directional shift across the four time points (Figure 3E). Notably, however, these shifts in differentiation trajectories between S1 and S2/S3 did not appear to be fully recapitulated in the observed temporal changes in ASPC subcluster proportions.

Our data demonstrated a significant increase in the proportion of S1 following 2 weeks of cold stimulation at 4°C (Figures 3F, G). This finding contrasts with previous reports by Liu et al. (2023) who observed a decrease in ASCs and a concomitant increase in preAs and Aregs under cold conditions. This discrepancy may be attributed to variations in the intensity and duration of cold stimulation between the studies, suggesting that prolonged and lower-temperature cold stimulation might activate the proliferative capacity of S1, thereby driving its transient increase in proportion. Upon return to thermoneutrality for 2 weeks, the proportions of S1, S2, and S3 largely reverted to their pre-cold stimulation levels, likely a consequence of the aforementioned shifts in differentiation trajectories. Furthermore, we observed a consistent increase in the proportion of S6 throughout the experimental time course. In contrast, the immune-related subclusters S4 and S5 exhibited distinct temporal dynamics: S4 initially increased and subsequently decreased, while S5 displayed the inverse pattern. However, the proportion of S4 consistently surpassed that of S5 across all time points. Notably, these cellular compositional changes may play critical roles in promoting thermal transition-induced iWAT remodeling.

### 3.4 Reprogramming of adipocyte subpopulations in iWAT upon return to thermoneutrality after cold stimulation

**FIGURE 4:**
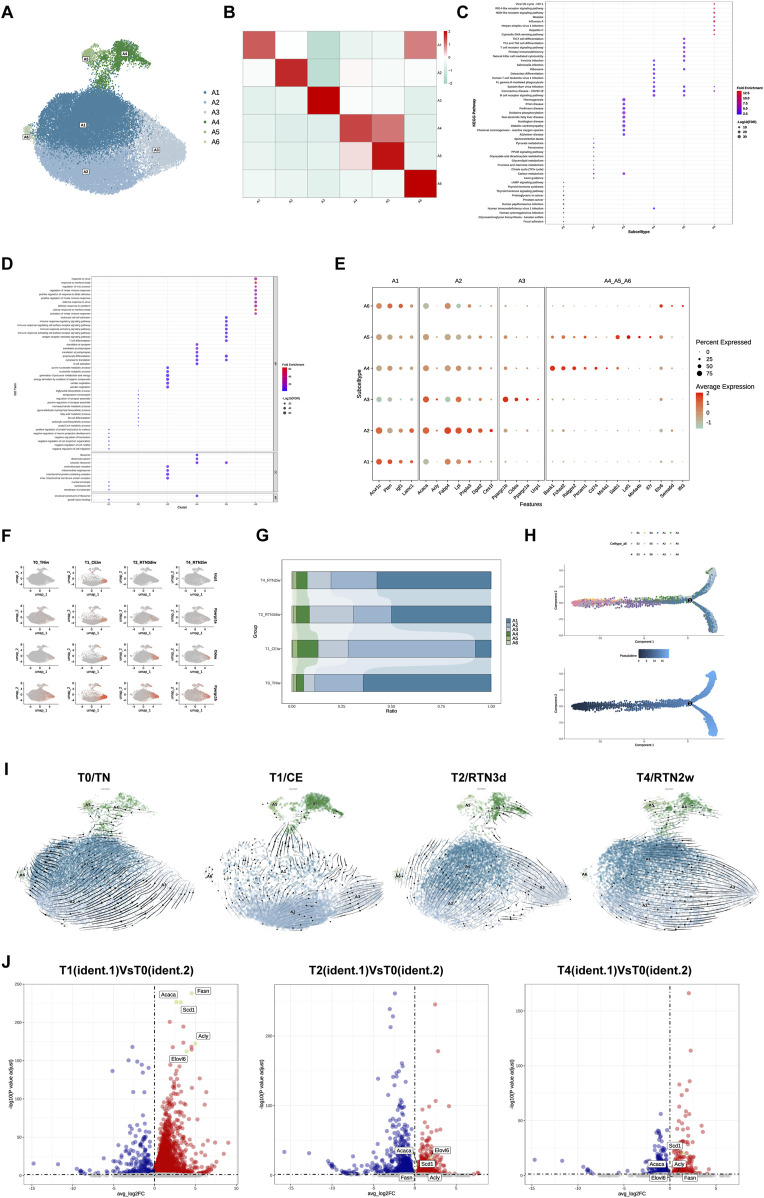
Reprogramming of Adipocyte Subpopulations in iWAT Upon Return to Thermoneutrality After Cold Stimulation. **(A)** UMAP of subclusters (A1-A6) identified within the adipocytes following re-clustering. **(B)** Heatmap showing the average scaled gene module scores for the top 50 most significant genes within each adipocyte subpopulation (A1-A6). **(C)** KEGG pathway and **(D)** GO enrichment analyses of differentially expressed genes across adipocyte subpopulation (A1-A6). The top 10 enriched pathways/terms are shown for comparison. **(E)** Bubble plot showing the expression of characteristic marker genes within adipocyte subpopulations (A1-A6). **(F)** Feature plots showing the expression of browning markers *Ucp1, Ppargc1α, Ppargc1β,* and *Cidea* in subcluster A3 across the T0, T1, T2, and T4. **(G)** Percent bar graph showing the changes in the composition of adipocyte subpopulatins (A1-A6) under T0, T1, T2, and T4. **(H)** Trajectory inference between ASPC subpopulations and adipocyte subpopulations. **(I)** RNA velocity analysis of adipocyte subclusters (A1-A6) across the T0, T1, T2, and T4. **(J)** Volcano plots showing the expression of DNL-related genes (*Acly, Acaca, Fasn, Elovl6,* and *Scd1*) in subcluster A3 at T1, T2, and T4 relative to T0. Upregulated genes (average logFC >0 and adjusted p-value <0.05) are shown in red, while downregulated genes (average logFC <0 and adjusted p-value <0.05) are shown in blue.

Numerous studies investigating mature adipocytes in AT across diverse anatomical depots in mammals have unequivocally demonstrated the existence of distinct subpopulations exhibiting heterogeneous functionalities (Corvera, 2021). These inherent functional disparities may be further amplified or modulated in response to external stimuli. To comprehensively delineate the adaptive reprogramming of mature adipocyte subpopulations within iWAT following cold stimulation and subsequent return to thermoneutrality, we performed re-clustering analysis, identifying 6 distinct adipocyte subpopulations: A1-A6 (Figures 4A,B). To further characterize the biological features and identify potential marker genes for each subpopulation, we conducted comprehensive KEGG and GO analyses of their differentially expressed genes (Figures 4C,D). Our analysis revealed that subcluster A1 exhibits a transcriptional program indicative of a regulatory role in a myriad of adipocyte biological processes. This subpopulation displayed elevated expressions of genes such as *Acvr1c*, *Pten*, *Igf1*, and *Lamc1*. Notably, KEGG analysis unveiled a pronounced enrichment for focal adhesion pathways, suggesting that A1 may serve as a pivotal function in mediating dynamic interactions between adipocytes and their surrounding microenvironment. A2 and A3 were both prominently associated with metabolic pathways, albeit with distinct functionalities. A2 exhibited a transcriptional signature characteristic of specialized lipid handling, with high expression of genes involved in lipid biosynthesis (*Acaca*, *Acly*) and fatty acid uptake, transport, and esterification (*Dgat2*, *Fabp4*, *Lpl*, *Pnpla3*, *Ces1f*) (Figure 4E). This transcriptional profile intriguingly aligns with the lipogenic adipocytes (LGAs) and lipid scavenger adipocytes (LSAs) previously identified in sWAT by Liu et al. (2023), suggesting that A2 may represent a functionally heterogeneous subpopulation. Conversely, A3 displayed enrichment in oxidative phosphorylation pathways, underscoring its involvement in active oxidative respiration and energy metabolism. Intriguingly, A4, A5, and A6 were all characterized by the expression of immune markers (including *Bank1, Cd74, Ms4a1, Il7r*) and enrichment in immune-related pathways, resembling the antigen-presenting adipocyte (APA) subpopulation identified by Liu et al. (2023) that potentially engages in MHC class II-mediated antigen presentation. This suggests their participation in diverse immune responses within the iWAT microenvironment and prompts us to classify A4, A5, and A6 as immune-related adipocyte subtypes.

Of particular interest, in alignment with Liu et al.’s identification of the thermogenic adipocyte subpopulation (BA) (Liu et al., 2023), our data also corroborate the prevailing paradigm that cold stimulation elicits the emergence of thermogenic beige adipocytes within iWAT, a phenomenon known as browning. Among the delineated adipocyte subpopulations (A1-A6), A3 uniquely exhibited a thermogenic transcriptional program. Not only were the differentially expressed genes in A3 significantly enriched in thermogenesis-related pathways, but the subtype also displayed robust expression of canonical browning markers, including *Ucp1*, *Ppargc1α*, *Ppargc1β*, and *Cidea* (Figure 4E). Furthermore, the temporal expression dynamics of these markers during cold stimulation and subsequent return to thermoneutrality mirrored the changes observed in UCP1 levels via IF staining (Figure 1D), exhibiting high expression during cold stimulation followed by a gradual decline upon rewarming, ultimately diminishing to negligible levels (Figure 4F). Furthermore, we observed that A3 exhibited significantly elevated expressions of *Atp5k, Atp5e, Coc7a1,* and *Lgr6* compared to other adipocyte subclusters (Supplementary Figure S1C). This expression profile is consistent with the recently reported characteristics of the P2 adipocyte subpopulation (Wang et al., 2024), which possesses a unique thermogenic capacity mediated by futile cycling, a mechanism independent of UCP1. This observation raises the intriguing possibility of functional heterogeneity within the A3 with respect to thermogenic mechanisms, suggesting that these cells may engage diverse thermogenic programs in response to cold challenges.

Analogous to the analysis of ASPCs subpopulations, we investigated the compositional dynamics of adipocyte subpopulations across the four experimental time points. Cold stimulation elicited an increase in the proportion of A3, the thermogenic adipocyte subpopulation endowed with the capacity to maintain core body temperature under such conditions. Concurrently, we observed the increase of A2, A4, and A5. However, upon return to thermoneutrality for 2 weeks, the proportions of all subpopulations underwent dynamic shifts, with the majority reverting to near pre-cold stimulation levels (Figure 4G). Interestingly, studies have demonstrated that cold-induced expansion of thermogenic adipocytes primarily arises from *de novo* differentiation from ASPCs (Berry et al., 2016) and transdifferentiation of existing white adipocytes (Cinti, 2009). In our study, trajectory inference analysis between A1-A6 and ASPCs revealed that ASPCs consistently occupied progenitor positions at the trajectory origin, while adipocyte subclusters resided at terminal positions. In addition, A1 appeared to be positioned closer to ASPCs in the trajectory compared to A2 and A3, suggesting that it may represent an earlier stage of mature adipocyte development (Figure 4H). RNA velocity analysis of A1-A6 across four time points further substantiated this notion, with A1 consistently positioned at the “origin” of adipocyte trans-differentiation (Figure 4I). Notably, the directionality of A1 transdifferentiation displayed condition-dependent plasticity. Before cold stimulation, A1 possesses the potential to transdifferentiate into A2, maintaining a relatively stable equilibrium between these two subpopulations that primarily mediate energy storage and ectopic lipid deposition prevention. During cold stimulation, the proportion of A1 decreased significantly, likely attributed to its transdifferentiation into A2, which subsequently exhibited a propensity to transdifferentiate into thermogenic A3, thereby enhancing iWAT thermogenic capacity. Furthermore, the A2→A3 transition within this multi-step transdifferentiation cascade (A1→A2→A3) appears to mirror the cold-induced conversion from LGAs to BAs reported in a prior study (Liu et al., 2023). Upon initial return to thermoneutrality, A3 experienced diminished thermogenic capacity concurrent with the gradual recovery of A1 proportions. Although A1→A2→A3 transdifferentiation persisted, we observed an emerging phenomenon of direct A1→A3 transdifferentiation. After 2 weeks of thermoneutral reacclimation, A3 completely lost thermogenic functionality, while A1 exhibited a tendency to bypass A2 and transdifferentiate directly into A3. Interestingly, within A3 at this time point, only *Scd1* remained upregulated among the *de novo* lipogenesis (DNL)-related genes (*Acly, Acaca, Fasn, Elovl6,* and *Scd1*) that were significantly upregulated during cold stimulation (Figure 4J).

### 3.5 Dynamic intercellular communication networks in iWAT undergo remodeling upon return to thermoneutrality after cold stimulation

**FIGURE 5:**
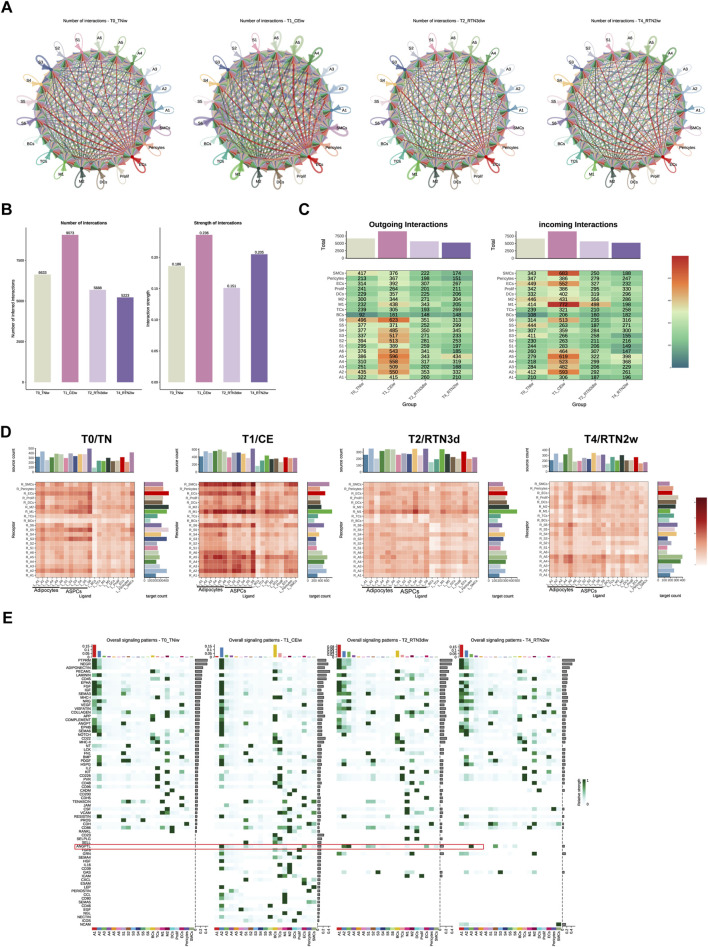
Dynamic Intercellular Communication Networks in iWAT Undergo Remodeling Upon Return to Thermoneutrality After Cold Stimulation. **(A)** Circle plots showing intercellular communication networks within iWAT at T0, T1, T2, and T4. **(B)** Bar graph showing the number and strength of intercellular communication interactions at T0, T1, T2, and T4. **(C)** Heatmap showing the number of outgoing/incoming signals for each cell type in iWAT at T0, T1, T2, and T4. The bar graph represents the sum of each column. **(D)** Heatmap showing the total number of potential ligand-receptor pairs identified by Cell Chat between different cell types in iWAT at T0, T1, T2, and T4. The bar graph represents the sum of each column or row. **(E)** Overall signaling patterns within iWAT, represented as the upregulated information flow in different cell types at T0, T1, T2, and T4.

Cell-cell communication (CCC) constitutes a fundamental attribute of multicellular organisms, orchestrating a myriad of critical biological processes, including cellular metabolism, energy conversion, growth regulation, development, and immune responses, through intricate and dynamic communication networks established between cells (Su et al., 2024). To comprehensively elucidate the contributions of potential ligand-receptor interactions to the coordinated and dynamic remodeling of iWAT during cold stimulation and subsequent return to thermoneutrality, we leveraged CellChat software and the CellphoneDB database to systematically analyze intercellular communication networks across four distinct time points (T0, T1, T2, and T4). This comprehensive analysis revealed a repertoire of 26,617 significant protein ligand-receptor pairs that mediate intercellular communication within the iWAT microenvironment. Subsequently, we utilized the identified ligand-receptor pairs to construct comprehensive intercellular communication networks encompassing all cell types at each time point (Figure 5A). Consistent with the observations of Shamsi’s team (Shamsi et al., 2023) regarding enhanced intercellular communication in brown adipose tissue (BAT) during cold stimulation, we observed a marked amplification of CCC within iWAT following 2 weeks of continuous cold stimulation. However, upon return to thermoneutrality, both the number and strength of CCC exhibited a discernible decline (T0: 6633, T1: 9073, T2: 5688, T4: 5233; Figure 5B).

Analysis of ligand and receptor expression profiles across cell populations at the four time points (Figures 5C,D) revealed that adipocytes and ASPCs exerted a dominant influence on intercellular signaling during cold stimulation, while M1 and SMCs exhibited a greater receptivity to incoming signals. This pattern underwent a gradual shift upon return to thermoneutrality. For instance, the receptivity of SMCs to incoming signals was attenuated by 3 days post-rewarming, while adipocytes emerged as the predominant orchestrators of intercellular communication, exhibiting both high signal transmission and reception, by 2 weeks post-rewarming. Overall, adipocytes consistently occupied a central position within the intercellular communication landscape throughout the cold stimulation and rewarming phases, maintaining robust communication with ASPCs, various immune cell populations, and endothelial cells. Cold stimulation potentiated these interactions, while return to thermoneutrality elicited a gradual attenuation of these connections, presumably to facilitate the adaptive remodeling of iWAT structure and function in response to evolving environmental demands.

To further dissect the intricate communication patterns between adipocytes and other cell types during cold stimulation and subsequent return to thermoneutrality, we undertook a thorough investigation of the signaling pathway landscapes across T0, T1, T2, and T4 (Figure 5E). The results indicated that A1, A2, and A3, the subpopulations of adipocytes, exhibited a pronounced susceptibility to environmental temperature fluctuations, as evidenced by the marked remodeling of their signaling pathway profiles across the experimental conditions. Based on the distinct characteristics and temporal dynamics of these shifts, two broad categories of signaling pathways emerged. The first category encompassed pathways inherent to the A1 subpopulation under thermoneutral conditions. These pathways, including PTPRM, NEGR, ADIPONECTIN, LAMININ, EPHA, FGF, IGF, SEMA3, VEGF, COMPLEMENT, ANGPT, EPHB, and SEMA6, exhibited robust signaling activity in A1 prior to cold stimulation (T0), which was subsequently attenuated or abrogated upon cold stimulation. Concomitantly, these pathways displayed heightened activity in A2 during cold stimulation, ultimately reverting to A1 upon return to thermoneutrality. This dynamic interplay suggests a potential “compensatory phenomenon” in which A2 temporarily assumes the signaling role intrinsic to A1 during cold acclimation. The second category comprised pathways that were quiescent in all adipocyte subpopulations at T0 but were activated in response to sustained cold stimulation. These pathways, including ANGPTL, TGFβ, GRN, HGF, CD39, LEP, PERIOSTIN, CCL, CD80, CD46, EGF, and NECTIN, were largely restored to their pre-cold stimulation levels upon rewarming, with the notable exception of the ANGPTL pathway in A3, which maintained elevated signaling activity. The sustained activation of ANGPTL signaling in A3 may reflect a prolonged adaptation to cold, potentially contributing to the maintenance of a thermogenic phenotype even after the cessation of the cold stimulus. In fact, despite the distinct temporal dynamics and cellular mediators associated with these two categories of pathways, they shared involvement in angiogenesis, inflammatory responses, and cellular development and differentiation. These processes are likely crucial for the structural and functional adaptations of adipocytes during cold stimulation and rewarming. Previous studies (Xue et al., 2009) have demonstrated that adipose tissue remodeling and expansion involve a physiological inflammatory response, encompassing various cellular differentiation and developmental processes that necessitate alterations in the vascular network to ensure adequate oxygen and nutrient supply.

### 3.6 Extensive intercellular communication with adipocyte subpopulation A3 coordinates thermogenic program activation and deactivation

**FIGURE 6:**
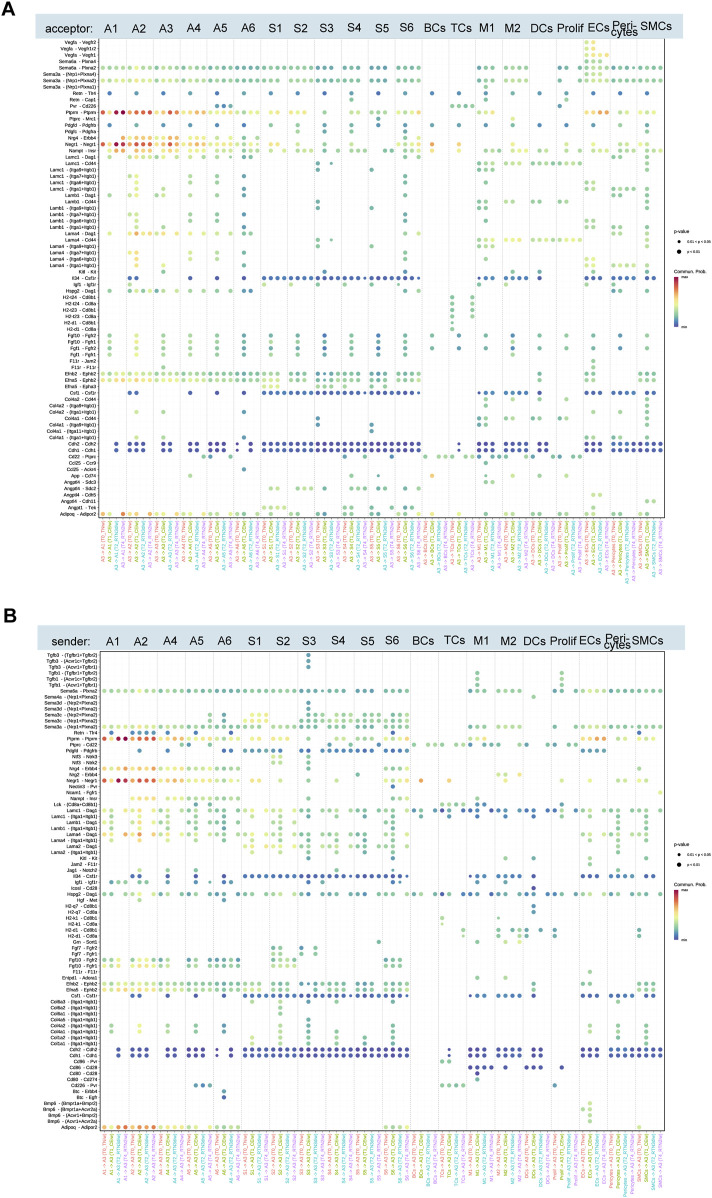
Ligand-receptor pairs mediated by the A3 subset upon cold stimulation and return to thermoneutrality for 2 weeks. **(A,B)** Upregulated ligand-receptor pairs between adipocyte subcluster A3 and other adipocyte subclusters, ASPC subclusters, immune cells, and angiogenesis-related cells at T0, T1, T2, and T4.

As described above, the thermogenic capacity of adipocyte subpopulation A3 is activated during cold stimulation and subsequently declines upon return to thermoneutrality. To understand the intercellular communication dynamics underlying this transition, we mapped the interactions between A3 and other adipocyte subpopulations, several immune cells, and cells involved in adipogenesis and angiogenesis (ASPCs, ECs, etc.) across the four time points (Figures 6A,B). During cold stimulation, A3 secreted *Retn, Pdgfd, Fgf10,* and *Fgf1*, which interacted with *Tlr4, Pdgfrβ, Fgfr1*, and *Fgfr2* on various cell types, increasing intercellular communication. These interactions were diminished upon return to thermoneutrality, suggesting that the remodeling of A3 during cold stimulation (proliferation and acquisition of thermogenic function) requires coordinated intercellular communication.

Overall, the interactions between A3 and other adipocytes, immune cells, and cells involved in adipogenesis and angiogenesis dynamically changed throughout the cold stimulation and rewarming phases, reflecting the adaptation to varying environmental temperatures. Most cold-induced changes gradually reverted to initial levels at different rates upon return to thermoneutrality, while others persisted. For instance, the interaction between A3 and A1/A2, mediated by the ligand-receptor pairs Ptprm-Ptprm, Negr1-Negr1, and Adipoq-Adipor2, showed significant changes in strength across all time points. However, the interaction between A3 and A1 was characterized by decreased signaling strength during cold stimulation and recovery upon rewarming, whereas the interaction between A3 and A2 showed increased signaling strength during cold stimulation, which persisted even after rewarming. *Ptprm*, a positive regulator of adipocyte differentiation, exhibits increased expression during 3T3-L1 adipogenesis (Lu et al., 2023). *Negr1* and *Adipoq* are implicated in cholesterol transport, lipid storage, and fatty acid oxidation (Mao et al., 2006; Yamauchi et al., 2007; Kim et al., 2017). Cold stimulation also activated the interaction between the receptor complex Itaga7/Itagb1, Itaga6/Itagb1, and Itaga1/Itagb1 on A2 and laminin family members (*Lamc1, Lamb1, and Lama1*) on A3, which was deactivated after 3 days of rewarming. Similarly, cold stimulation induced the interaction between amyloid precursor protein (APP) on A3 and *Cd74* on B cells, macrophages, and dendritic cells. Stimulation of CD74 has been shown to trigger signaling cascades that promote cell proliferation and survival (Su et al., 2017). This interaction was also deactivated after 3 days of rewarming.

Furthermore, our investigation into the interplay between A3 and cells integral to adipogenesis and angiogenesis, including ASPCs, endothelial cells, etc., we uncovered that cold stimulation significantly potentiated the interaction mediated by the binding of several collagen subtypes (*Col6a1, Col6a2, Col4a2, Col1a1, etc.*) to the integrin subunits *Itga1/Itgb1*. Intriguingly, a subset of interactions, specifically those involving the ligand *Angptl4* and its cognate receptors *Sdc2, Cdh11,* and *Cdh5*, exhibited a protracted deactivation profile. These interactions persisted even after a 3-day return to a thermoneutral environment, with some remaining evident for up to 2 weeks. This sustained activation suggests a potential role for *Angptl4* in the long-term cellular adaptation to cold stimulation. This observation is particularly noteworthy given the established role of *Angptl4* in modulating glucose and lipid metabolism (Xu et al., 2005; Robciuc et al., 2011). Moreover, *Angptl4* is a key transcriptional target of the glucocorticoid receptor (GR), a critical regulator of beige adipocyte whitening in thermoneutral conditions (Roh et al., 2018).

## 4 Discussion

sWAT, the largest and most plastic WAT depot, exhibits a remarkable capacity for remodeling in response to cold environments, notably through the generation of thermogenic adipocytes. This inherent plasticity has positioned sWAT as a promising therapeutic target for enhancing cold tolerance and ameliorating obesity, metabolic disorders, and even cancer (Cohen et al., 2014; Hanssen et al., 2015; Stine et al., 2016; Seki et al., 2022; Jurado-Fasoli et al., 2024). However, the direct clinical application of cold stimulation is hindered by practical limitations. Furthermore, and crucially, cold-induced thermogenesis is transient, diminishing upon return to thermoneutral conditions. Consequently, for humans who predominantly inhabit thermally controlled environments (i.e., thermoneutral conditions), understanding the mechanisms underlying the consolidation and maintenance of this thermogenic response is of paramount importance. While current research predominantly focuses on the effects of cold stimulation on WAT, the dynamic changes occurring within sWAT upon return to thermoneutrality remain poorly characterized. To address this knowledge gap, we established a comprehensive animal model encompassing both cold stimulation and subsequent return to a thermoneutral environment. Employing snRNA-seq as our primary investigative tool, we systematically characterized the iWAT transcriptome at key time points during the rewarming process. This approach facilitated a comprehensive and in-depth understanding of the cellular and structural remodeling events that occur within iWAT upon return to thermoneutrality. Moreover, our dataset constitutes a valuable public resource for elucidating the complex cellular and molecular mechanisms governing iWAT physiology and adaptation.

Consistent with previous studies, our analysis of iWAT during cold stimulation revealed the emergence of beige adipocytes, characterized by multilocular lipid droplets and high UCP1 expression. However, upon return to thermoneutrality, these beige adipocytes progressively declined, and by 2 weeks, the histological landscape resembled the pre-cold stimulation state, with unilocular white adipocytes predominating. Similarly, the average transcriptional levels of differentially expressed genes reverted to pre-cold stimulation levels after 2 weeks of rewarming. Despite this apparent reversion, previous work by Matthias (Rosenwald et al., 2013) et al. demonstrated that beige adipocytes persist within WAT even after returning to thermoneutrality, retaining the capacity to reactivate their beige phenotype upon subsequent cold stimulation. This observation aligns with the concept proposed by Feil (Feil and Fraga, 2012) et al. that epigenetic modifications, rather than alterations in cellular identity, may underlie this phenotypic plasticity. Our high-throughput sequencing data further revealed that the iWAT landscape following rewarming is not solely characterized by “whitened” beige adipocytes. A subset of cold-induced transcriptional changes persisted, while others reverted at varying rates. We propose that comparative analysis of these distinct reversion patterns and their underlying molecular mechanisms may offer valuable insights into strategies for mitigating WAT, and potentially BAT, whitening.

Beige adipocytes arise from diverse cellular origins, contributing to their heterogeneity. Lineage tracing studies (Cinti, 2009; Barbatelli et al., 2010; Lee et al., 2015) employing Adipoq-creERT2 mice have indicated that beige adipocytes within WAT may originate from the trans-differentiation of white adipocytes. Conversely, other studies suggest that beige adipocytes arise *de novo* from progenitor cells residing within the SVF(Wang et al., 2013; Berry et al., 2016). Our findings suggest that these two biogenic mechanisms are not mutually exclusive and may operate in concert, forming a continuum from *de novo* differentiation to trans-differentiation, with the S1 adipocyte subpopulation as the origin and A3 as the terminus. This hypothesis is largely consistent with the ASC→LGA→BA differentiation pathway proposed by Liu’s team (Liu et al., 2023). The difference lies in that our findings suggest the potential existence of an even earlier adipocyte state, A1, before the LGA (corresponding to A2 in our study) → BA (corresponding to A3) transdifferentiation, indicating a potentially complex multi-stage process where the A2 subpopulation serves as an intermediate transitional state in the A1→A3 conversion. However, this phenomenon may be linked to the thermogenic capacity of A3. Upon return to thermoneutrality for 2 weeks, a period characterized by diminished thermogenic gene and protein expression within A3, A1 adipocytes can transdifferentiate directly into A3. Within the A3 subpopulation, the expression of genes associated with DNL, including *Acly, Acaca, Fasn, Elovl6,* and *Scd1*, is significantly upregulated during cold stimulation. However, upon return to thermoneutrality, the expression of these genes progressively declines, with only *Scd1* expression persisting at the 2-week time point. This observation aligns with the findings of Lundgren et al. (2023) who performed bulk RNA sequencing on BAT following acute cold stimulation and subsequent return to thermoneutrality. Their study further demonstrated that the reactivation of the thermogenic program in BAT upon re-stimulation to cold is dependent on *de novo* lipogenesis within brown adipocytes. Specifically, knockdown of key DNL regulators, *Scap* and *Fasn*, in BAT significantly impaired cold tolerance upon secondary, but not initial, cold stimulation. In light of these findings, it is imperative to investigate whether *de novo* lipogenesis within the A3 adipocyte subpopulation plays an analogous role in the reactivation of the thermogenic program in WAT upon secondary cold stimulation. This line of inquiry holds a substantial promise for elucidating the regulatory mechanisms governing beige adipocyte plasticity and thermogenic capacity.

Beyond transdifferentiation, our trajectory inference analyses suggest a profound impact of environmental temperature on WAT plasticity through the potential induction of dedifferentiation. While re-stimulation to thermoneutrality following cold stimulation did not perturb the overall trajectory of A1 to A3 trans-differentiation, a marked shift was observed in the differentiation trajectory of ASPCs. Specifically, S2 and S3 cells, typically characterized by a higher degree of differentiation and the capacity to differentiate into mature adipocytes, appeared to undergo dedifferentiation into S1, which possesses a lower differentiation state and multi-lineage potential. This phenomenon of dedifferentiation has been documented in diverse physiological and pathological contexts, including skin fibrosis at wound sites (Zhang et al., 2019), mammary gland remodeling during lactation (Wang et al., 2018), and liposarcoma development (Thway, 2019). Recently, a study (Zhu et al., 2022) reported that during breast tumor progression, mammary adipocytes undergo dedifferentiation, relinquish their original cellular identity, and transform into smooth muscle-like cells and macrophage-like cells, contributing to tumor cell proliferation through immune modulation, inflammation, and extracellular matrix remodeling. Although these dedifferentiated cells exhibit distinct metabolic profiles compared to tumor-associated SMCs, they exert similar effects on tumor cell proliferation. While, to our knowledge, no prior reports have documented environmental temperature-induced dedifferentiation in adipogenic cells, our computational findings provide suggestive evidence for this phenomenon. We propose, based on these inferences, that re-stimulation to thermoneutrality may trigger the putative differentiation of adipose progenitor cells, generating multipotent mesenchymal stem cells that contribute to the maintenance of WAT homeostasis by promoting the formation of diverse cell types beyond mature adipocytes. This intriguing observation warrants further investigation and represents a crucial avenue for future research to fully delineate the intricate interplay between environmental temperature and WAT plasticity. However, it is important to emphasize that these observations are based on computational inferences from transcriptomic snapshots and require direct experimental validation to confirm these lineage relationships and differentiation dynamics.

As underscored by our findings, tissue homeostasis is intricately orchestrated through complex intercellular communication networks. Recent high-throughput sequencing studies have illuminated the extensive crosstalk among diverse cell types during cold-induced AT remodeling, encompassing both WAT and BAT (Liu et al., 2023; Shamsi et al., 2023). Notably, Shamsi et al. (2023) demonstrated that cold stimulation potentiates intercellular communication within BAT and further emphasized the multifaceted roles of adipogenic progenitors, extending beyond adipogenesis to encompass processes such as immune modulation and angiogenesis. Similarly, our constructed cell-cell communication network revealed that cold stimulation elicits more extensive interactions among various cell types within iWAT. However, upon re-stimulation to thermoneutrality, these interactions exhibit a progressive decline in both number and strength. Notably, adipocytes consistently occupy a central position within these intercellular communication networks. Analysis of signaling pathway dynamics within adipocyte subpopulations (A1, A2, and A3) across the four time points revealed that A1, A2, and A3 undergo the most pronounced changes. Furthermore, the shift in environmental temperature induces dynamic modulation of these signaling pathways, affecting their activation status and the cell types involved. These pathways are primarily associated with immune responses, angiogenesis, and cell differentiation and development, ensuring adaptive remodeling of the iWAT microenvironment in response to prevailing environmental conditions. Interestingly, the majority of these signaling patterns ultimately revert to their pre-cold stimulation state, albeit at varying rates, with the notable exception of the ANGPTL pathway. Notably, this pathway remained active in thermogenic A3 adipocytes even after 2 weeks of rewarming to thermoneutrality, a period characterized by a substantial reduction in thermogenic markers. The ligand *Angptl4* binds to its cognate receptors (*Sdc2*, *Cdh11*, and *Cdh5*) to promote interactions between A3 and cells involved in adipogenesis and angiogenesis. *Angptl4*, a recently identified adipokine, exerts pleiotropic effects on glucose and lipid metabolism, improves insulin resistance, and is implicated in various risk factors for heart failure, including obesity, diabetes, and coronary heart disease (Xu et al., 2005; Kim et al., 2010; Lichtenstein et al., 2010; Liu et al., 2019). Thus, the sustained activation of the ANGPTL pathway in rewarmed iWAT raises the intriguing possibility of complex inter-organ crosstalk. Moreover, this persistent ANGPTL signaling offers another interesting perspective: it might facilitate the transition of thermogenic adipocytes towards a ‘whitened’ state following the cessation of the cold stimulus. However, this remains a hypothesis necessitating further investigation. In this context, the GR may represent a critical entry point, given that *Angptl4* is a prominent transcriptional target of GR. Concurrently, previous studies (Roh et al., 2018) have indicated that GR mediates the “whitening” of beige adipocytes in thermoneutral environments, and adipocyte-specific GR knockout mice exhibit delayed beige adipocyte whitening upon return to thermoneutrality following cold stimulation. Therefore, the sustained ANGPTL signaling we observed may be at least partially mediated by GR activity in the post-rewarming phase. Undeniably, subsequent studies are warranted to thoroughly investigate whether *Angptl4* serves as a key downstream effector of GR in promoting and/or maintaining the ‘whitened’ phenotype of A3 adipocytes, thereby elucidating whether the ANGPTL pathway actively participates in sustaining the ‘whitened’ state and its interplay with GR signaling in this process. Collectively, investigating *Angptl4* as a strategic entry point for consolidating beige adipocyte thermogenic identity and preserving multi-organ metabolic activity within a thermoneutral environment may yield valuable insights for future research. This study acknowledges the limitations in fully exploring this intricate interplay, necessitating further experimental and dedicated research endeavors to elucidate the underlying mechanisms and broader implications. Subsequent inquiries should rigorously incorporate advanced molecular techniques, such as CRISPR-Cas9-mediated knockout, siRNA interference, and/or targeted gene overexpression, to establish definitive causal relationships and validate biological significance. Crucially, while the single-cell transcriptomic evidence presented herein offers insightful perspectives into the dynamic remodeling of sWAT in response to ambient temperature shifts at a micro-level, it requires substantial complementary biological validation. Our future research trajectory will adopt a multidimensional validation framework, employing fluorescence-activated cell sorting (FACS) and genetic lineage tracing to isolate distinct cell subpopulations, coupled with relevant animal models and potentially clinical samples, to conduct in-depth validation. This approach aims to establish a translational continuum from mechanistic exploration to therapeutic hypothesis generation.

## 5 Conclusion

In this study, we established a comprehensive animal model encompassing cold stimulation and subsequent return to thermoneutrality. Utilizing morphological analysis, functional protein expression profiling, and transcriptomic analysis, we identified the 2-week time point following re-stimulation to thermoneutrality as a critical juncture in the iWAT adaptive process. We generated a single-cell resolution atlas of iWAT during this rewarming period, enabling detailed characterization of cellular remodeling events. Our investigation focused on the dynamic changes within the ASPC and adipocyte compartments of iWAT, encompassing their composition, gene expression profiles, and differentiation trajectories. Notably, we predicted a previously unreported phenomenon: re-stimulation to thermoneutrality induced dedifferentiation within the ASPC compartment, contributing to the partial reversion of cold-induced cellular composition changes. Furthermore, we inferred distinct transdifferentiation pathways for thermogenic adipocyte subpopulations during cold stimulation and subsequent rewarming. Analysis of our constructed cell-cell interaction network revealed intricate communication patterns among various cell types throughout the 2-week rewarming period, highlighting the central role of adipocytes as key mediators of intercellular crosstalk. We further identified and compared signaling pathway dynamics within the A1, A2, and A3 adipocyte subpopulations, which exhibited the most pronounced changes. Finally, we explored the interactions between A3 and other cell types during both the activation and deactivation of the thermogenic program, offering insights into the underlying mechanisms. These findings may provide a foundation for identifying novel targets to enhance adaptive thermogenesis and develop innovative therapeutic strategies for obesity and metabolic disorders.

## Data Availability

The datasets presented in this study can be found in online repositories. The names of the repository/repositories and accession number(s) can be found below: https://www.ncbi.nlm.nih.gov/ with the accession number PRJNA1188547.
