# Supplementary material for: snRNA-seq reveals subcutaneous white adipose tissue remodeling upon return to thermoneutrality after cold stimulation
Source: Front Cell Dev Biol. 2025 May 22;13:1578180. doi: 10.3389/fcell.2025.1578180 (PMC12138199; doi:10.3389/fcell.2025.1578180)
Supplement: Supplementary file 1 [file DataSheet1.docx]

Supplementary Material

# Supplementary Figures

**
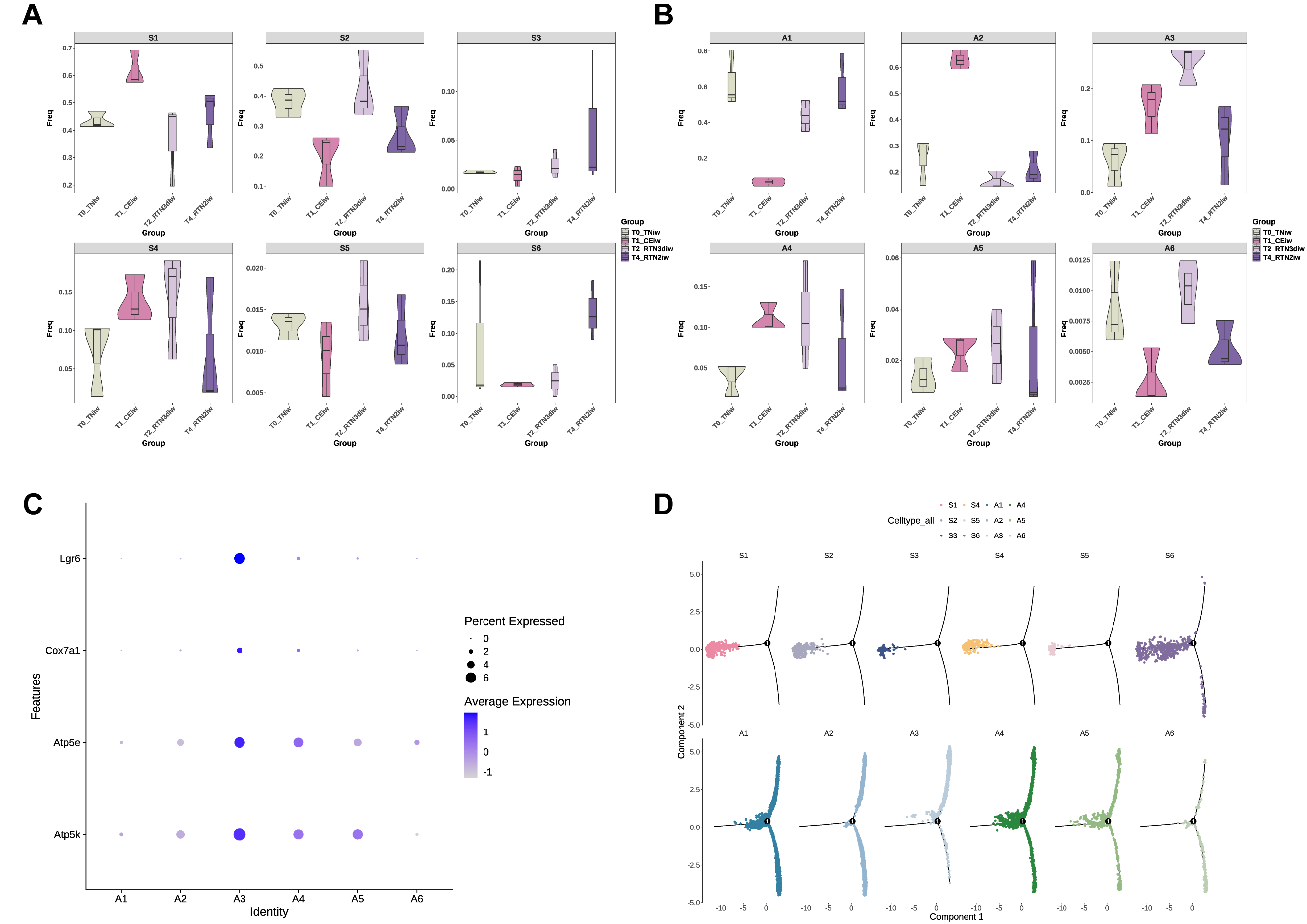
**

**Supplementary Figure 1.** **Subclustering analysis of ASPCs and adipocytes**

**(A)** Boxplots showing the composition of ASPC subpopulations at T0, T1, T2, and T4. **(B)** Boxplots showing the composition of adipocyte subpopulations at T0, T1, T2, and T4. **(C)** Bubble plot showing the significant expression of Atp5k, Atp5e, Cox7a1, and Lgr6 in subcluster A3. **(D)** Trajectory inference of ASPC and adipocyte subpopulations.
